# Factors affecting deer ked (*Lipoptena cervi*) prevalence and infestation intensity in moose (*Alces alces)* in Norway

**DOI:** 10.1186/1756-3305-5-251

**Published:** 2012-11-12

**Authors:** Knut Madslien, Bjørnar Ytrehus, Hildegunn Viljugrein, Erling J Solberg, Kent Rudi Bråten, Atle Mysterud

**Affiliations:** 1 Norwegian Veterinary Institute, Oslo, Norway; 2 Norwegian Institute of Nature Research, Oslo, Norway; 3 Hedmark University College, Campus Evenstad, Evenstad, Norway; 4 Centre for Evolutionary and Ecological Synthesis, University of Oslo, Oslo, Norway

**Keywords:** Bogs, Deer ked, GIS, Habitat, Latitude, Longitude, Moose density, Prevalence, Infestation intensity, Scots pine

## Abstract

**Background:**

The deer ked (*Lipoptena cervi*), a hematophagous ectoparasite of Cervids, is currently spreading in Scandinavia. In Norway, keds are now invading the south-eastern part of the country and the abundant and widely distributed moose (*Alces alces*) is the definitive host. However, key factors for ked abundance are poorly elucidated. The objectives of our study were to (i) determine deer ked infestation prevalence and intensity on moose and (ii) evaluate if habitat characteristics and moose population density are determinants of deer ked abundance on moose.

**Methods:**

In order to identify key factors for deer ked abundance, a total of 350 skin samples from the neck of hunted moose were examined and deer keds counted. Infestation intensity was analyzed in relation to moose age and sex, moose population density and landscape characteristics surrounding the killing site.

**Results:**

Deer ked infestation prevalence was 100%, but infestation intensity varied from 0.001 to 1.405 keds/cm^2^. Ked intensity was highest in male yearlings (~1.5 years) and positively associated with longitude and Scots pine (*Pinus sylvestris*) dominated habitat and negatively associated with bogs and latitude. Moose population density during autumn showed a tendency to be positively associated, while altitude tended to be negatively associated with ked intensity.

**Conclusions:**

Deer keds exploit the whole moose population within our study area, but are most prevalent in areas dominated by Scots pine. This is probably a reflection of Scots pine being the preferred winter browse for moose in areas with highest moose densities in winter. Ked intensity decreases towards the northwest and partly with increasing altitude, probably explained by the direction of dispersal and reduced temperature, respectively. Abundant deer ked harm humans and domestic animals. Moose management authorities should therefore be aware of the close relationship between moose, deer ked and habitat, using the knowledge as a management tool for locally regulating the ked burden.

## Background

Parasitism is very widespread, and defined as: “an ecological association between species in which the parasite lives on or in the body of the other, the host and the relationship is usually regarded as obligatory for the parasite and harmful or damaging for the host” [1]. With recent climate change, understanding the ecology of ectoparasites has become topical, as their niches will change geographically and some are vectors of disease [2]. In much of North America and Europe, populations of cervids have increased markedly in abundance and distribution, and this will likely affect the level of ectoparasitism in these species [3].

The deer ked (*Lipoptena cervi*) is a hematophagous ectoparasite of cervids in Europe, Asia, Africa and North-America [4]. The deer ked invaded south-eastern Norway from Sweden with the first case reported in 1983 [5] and rapidly spread north- and westwards [6]. By 1999, the distribution range embraced large parts of south-eastern Norway [6]. During the autumn swarming period, the deer keds may attack a variety of animals and humans, but only cervids seem to be able to function as definitive hosts [4]. In continental Europe, roe deer (*Capreolus capreolus*) is the most important, definitive host for deer keds [4], whereas in Norway, the abundant and widely distributed moose (*Alces alces*) [7] fill this role [8]. Hunters report that deer keds have become highly abundant on harvested moose in south-eastern Norway [6]. These observations are further supported by people using forest areas for recreation, complaining about the increasing number of attacking keds during autumn. Furthermore, a severe outbreak of alopecia in moose was attributed to severe deer ked infestation in Norway and Sweden in 2006–2007 [9].

However, knowledge about extrinsic factors that determine deer ked abundance are poorly described in the literature, though mild temperatures, especially during autumn [9] and high moose population density [10] have been suggested to be favorable for deer ked survival and host acquisition.

The life cycle for deer keds is peculiar. After landing on the host, the insect crawls into the coat, sheds its wings and engorges itself with blood, soon followed by copulation and deposition of prepupae that immediately pupate and passively fall onto the ground [4]. Consequently, pupae are dropped from the moose coat, whenever they walk or lay in bedding sites, through the late autumn and winter. The pupae then remain on the ground until August-September, when they emerge as winged, adult keds. Keds are short-distance flyers [11,12], which implies that the spatial distribution and density of winged keds would be highly dependent on the distribution of pupae, although predation by birds and rodents may to some extent interfere with the direct relationship between the number of pupae voided and the number of emerged adults [13,14]. As the pupae are dispersed from the moose coat, this in turn relies on the spatial use of habitat by the infested animals. The preferred diet of moose in late autumn and winter is primarily woody twigs of deciduous trees, shrubs and conifers [15]. Based on amount of biomass ingested, Scots pine (*Pinus sylvestris*) and birches (*Betula* spp.) seem most important in the study area during winter [16].

In consideration of the above mentioned extrinsic factors and the biology of deer ked, we hypothesize that the deer ked infestation intensity in moose is highest in: 1) selected winter habitats (e.g. Scots pine) and 2) areas with high moose population density. In addition, as the deer ked was first reported in the southernmost part of the study area in 1985, and was in 1991–1992 reported in the northern and western edge of the study area [5], the deer ked population in the northwest may still be in an expansion phase and may not have yet reached carrying capacity. Consequently, 3) a spatial, decreasing gradient in ked intensity on moose from southeast to northwest is also hypothesized.

The objectives of our study were to: i) determine deer ked infestation prevalence and intensity on moose in a typical deer ked biotope in south-eastern Norway; ii) evaluate if landscape characteristics and moose population density act as determinants of deer ked abundance on moose.

## Methods

### Study area

**Figure 1:**
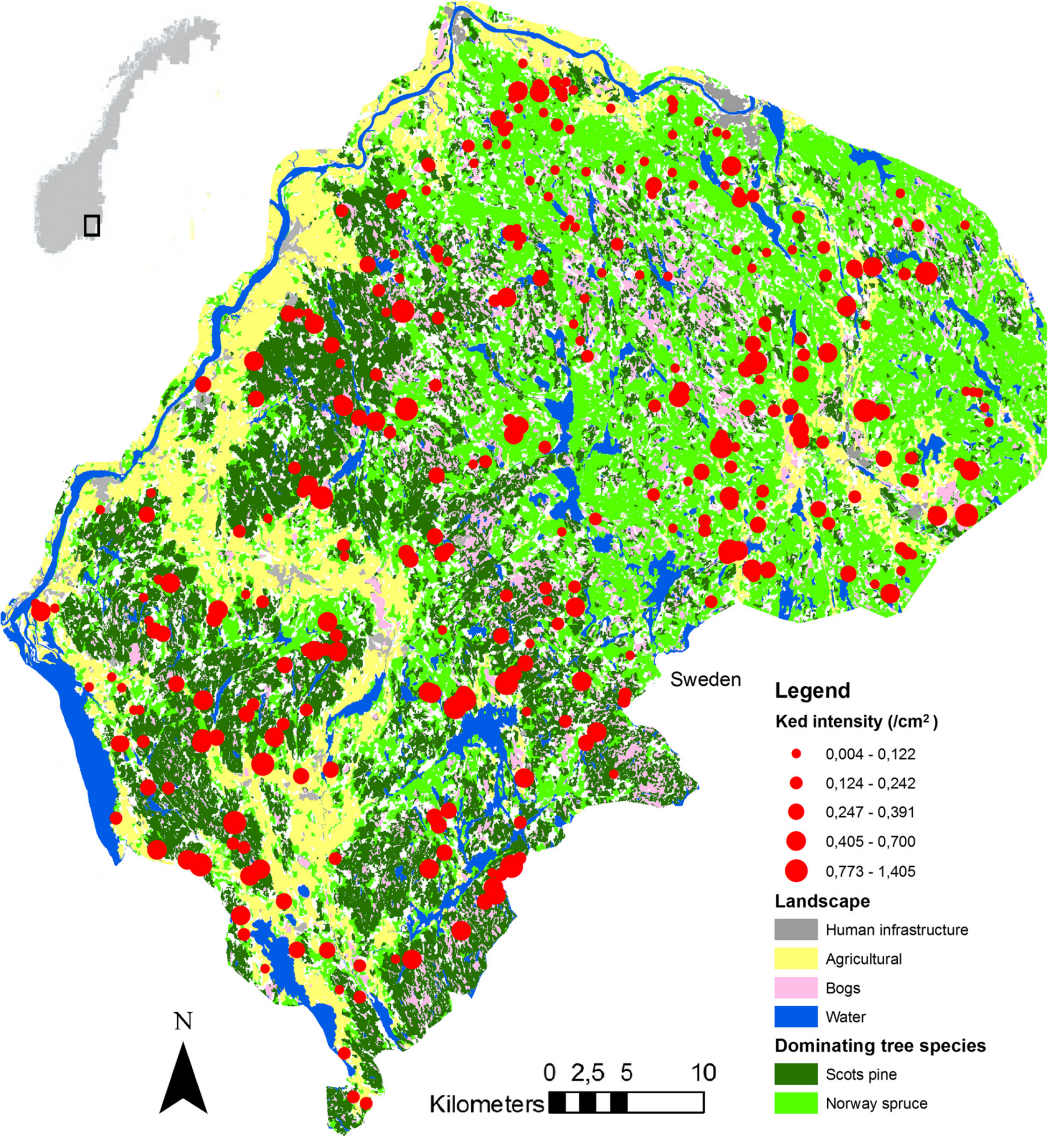
**Deer ked intensity in hunted moose in study area, South-Eastern Norway.** Red dot size reflects ked intensity and geographical position of killing site. Forests are mainly dominated by Scots pine and Norway spruce. White areas are coniferous or deciduous forests. See Methods for classification criteria.

The study area (2.097 km^2^) is in southern Norway (59°35^′^ - 60°15^′^ N - 11°09^′^ - 12°21^′^E), including parts of the counties of Akershus and Hedmark (Figure 1). The area ranges from about 100 meters above sea level (m.a.s.l.) in the southwest to 466 m.a.s.l. in the north, covering both the middle boreal, southern boreal and boreo nemoral vegetation zones [17]. The landscape is characterized by undulating, forest-covered hills with some farmland in intersecting valleys. Relatively rich farmland on marine deposits dominates the outskirts of the area in the south and west [17] and agricultural areas make up 12.4% of the total area [18]. The forested part is dominated by Scots pine, Norway spruce (*Picea abies*) and birch. In addition, grey alder (*Alnus incana*), aspen (*Populus tremula*), rowan (*Sorbus aucuparia*) and goat willow (*Salix caprea*) are found in lower densities in all parts of the forested area. The study area is identical to “Moose Region East” (ERRØ); a moose management organization of about 2.000 moose hunters in 188 hunting teams, harvesting about 1.400 moose annually. ERRØ is a well-managed organization which develops hunting plans, arranges meetings for the involved hunters, supports scientific research and facilitates communication between hunters, stakeholders and official wildlife management authorities. ERRØ is based on voluntary work. The deer ked has been present over the whole study area for at least a decade [6] and for nearly two decades in the southeastern part [5].

### Sample collection

We asked hunters in the study area to perform sampling from all harvested moose during the first week of hunting (October 5^th^- 12^th^) in 2010.

Sampling equipment and written instructions were distributed to all local hunting teams prior to the hunting season. The hunters were instructed to cut a 20 cm × 20 cm piece of skin from the neck, seal it in a plastic bag and store it at −20°C or a cool environment (+4°C), before submission to the Norwegian Veterinary Institute (NVI) for further analysis. Sampling was performed the same day as killing and before the carcass was cooled to avoid deer keds escaping from the coat.

Each skin sample was accompanied by a form with the following parameters included: Date of harvest, gender, age group and number of daily observed moose. Age group was, based on the hunters` assessment, classified into the following categories; calves (born same year ∼ 5–6 months old), yearlings (born the previous year ∼ 1.5 years old) and adults (> 2.5 years old). The location of the killing site was marked as a cross on a map printed on the back of the form. To estimate intensity of moose in the study area, we used a combination of harvest data and hunter moose observations. The “Moose Observation Monitoring” is a systematic sampling of number, age (calf, adult) and sex of all moose seen, as well as the number of hunter-days (hunting effort) conducted during the hunting season in Norway [19]. An index of population density, i.e. moose seen per hunter-day (SPUE) was determined from these data.

### Laboratory examination

In the laboratory, samples were thawed overnight in the plastic bag. The coat was cut close to the skin by an electric wool clipper (AlfaLaval CC320) and all loose hair, the inside of the plastic bag and naked skin was examined closely for presence of deer keds in particular and ectoparasites in general. All visible ectoparasites were counted. Length and width of skin samples were measured in order to calculate total skin area in each sample and deer ked infestation intensity (keds/cm^2^) was calculated.

### Habitat maps

We produced a digital map of the study area with five landscape categories: Human infrastructure, agricultural land, bogs, water and forest (Figure 1) [20]. Forest was further classified as either (1) “Scots pine” or (2) “Norway spruce” when any of these species covered > 50% of total forest volume, while designated as (3) “coniferous” when Scots pine and Norway spruce covered > 75% together and (4) “deciduous” when > 50% of birch and other deciduous tree species. Data on forest composition were downloaded from Sat-Skog [21]; a computerizing program which compiles field data from the National Forest Inventory [22] with digital topographic maps and satellite images. To analyze the potential effects of land cover on deer ked infestation in moose, we first calculated a buffer with a radius of 2 km around each killing site and quantified the land cover (m^2^) within each buffer. The moose population in the study area is non-migratory [23], and based on GPS locations for stationary moose in another Norwegian moose population [24], we estimated the average home range to be about 12.5 km^2^ during autumn. The buffer radius of 2 km was therefore designed to represent the theoretical, average home range (12.5 km^2^) of the moose study population during autumn, which coincides with the swarming period of keds. Buffers were created and map information extracted using the analysis tools in ArcMap 9.2 (ESRI, Redlands, CA, USA). For each killing site, we calculated the proportion of land cover within the buffer connected to the respective land cover categories.

### Data analysis

Generalized linear models (GLM) with normal error distribution were run to analyze the effect of explanatory variables on moose deer ked intensity [25]. Deer ked intensity was log-transformed in order to normalize the variance. The explanatory variables age class, sex and the age class-sex interaction was used in the basic model. Moose density, forest variables, proportion of area with bogs, altitude, latitude and longitude of killing sites were introduced in a forward selection approach, using the Akaike Information Criterion (AIC) for model selection (Table 1). Area of Norway spruce was strongly negatively correlated with area of Scots pine (Pearson correlation: -0.96) and was not included in the model. Area of Scots pine was negatively correlated with latitude and longitude (Pearson correlation: -0.50 and −0.65, respectively), while area of deciduous forest was negatively correlated with altitude (Pearson correlation: -0.58). All other covariables had correlations that were less than 0.50. Nonlinear relationships (as suggested by explorative analyses of spline-functions of the relevant variables) were accounted for by second order terms of the variables. All statistical analyses were performed in R version 2.14.1. [26].

**Table 1:** Forward model selection of variables explaining variation in ked intensity in moose

**Age**	**Gender**	**Age: gender**	**Moose density**	**Age: moose density**	**Altitude**	**Scotch pine**	**Norway spruce**	**Coniferous forest**	**Decidous forest**	**Bog**	**Longtitude**	**Latitude**	**(Latitude)2**	**AIC**	**dAIC**
x	x	x	x											945.4	65.5
x	x	x	x	x										948.5	68.6
x	x	x	x		x									937.4	57.5
x	x	x	x		x	x								915.7	35.8
x	x	x	x		x	x	x							917.3	37.4
x	x	x	x		x	x		x						913.8	33.9
x	x	x	x		x	x		x	x					915.2	35.3
x	x	x	x		x	x		x		x				912.9	33.0
x	x	x	x		x	x		x		x	x			893.8	13.9
x	x	x	x		x	x		x		x	x	x		887.9	8.0
**x**	**x**	**x**	**x**		**x**	**x**		**x**		**x**	**x**	**x**	**x**	**879.9**	0

The best model (dAIC=0) is highlighted in bold.

X = term included in model.

## Results

In total, 408 samples were received, corresponding to 62% of total moose harvested during the first week of the hunting season in the study area. However, 58 samples were rejected due to inadequate quality or inaccuracies in completing the form, resulting in a sample size of 350. The sample size consisted of 54 calves (21 males/33 females), 123 yearlings (78 males/45 females) and 173 adults (90 bulls/83 cows).

### Ectoparasite infestation prevalence and level of infestation intensity

Deer ked infestation prevalence (proportion of individuals in sample size infested by keds) was 100%. Between one and 487 keds were counted in each skin sample.

Skin sample infestation intensity (average number of keds/cm^2^) varied from 0.17 (male calves) to 0.31 (male yearlings) among sex and age groups (Figure 1).

Single nymphs of Castor bean ticks were found in three moose.

### Best model of deer ked infestation intensity

Deer ked intensity on moose was best predicted by a model including age, sex, age-sex interaction, moose density, altitude, area of Scots pine forest, area of coniferous forest, area of bogs, longitude and latitude (Table 2). No remaining pattern was seen in the residuals of the model.

**Table 2:** Parameter estimates of the best model predicting deer ked intensity in moose in south-eastern Norway

**Parameter**	**Estimate**	**SE**	***t***	***P***
Intercept	−1.436	0.106	−13.594	< 0.0001
Calf	−0.386	0.207	−1.865	0.063
Adult	−0.218	0.130	−1.684	0.093
Female	−0.146	0.158	−0.930	0.353
Moose density	0.362	0.233	1.558	0.120
Altitude	−0.00124	0.000756	−1.635	0.103
Area of Scots pine forest	0.982	0.281	3.497	0.001
Area of coniferous forest	1.664	0.965	1.725	0.085
Area of bogs	−21.8x10^6	9.21x10^6	−2.366	0.019
Latitude	−2.010	0.390	−5.157	0.004
Latitude^2	−6.472	2.069	−3.128	0.002
Longitude	0.529	0.243	2.176	0.030
Calf x Female	0.417	0.282	1.476	0.141
Adult x Female	−0.0860	0.203	−0.424	0.672

Intercept = male yearlings (~ 1.5 years) at average level of all continous covariables.

**Figure 2:**
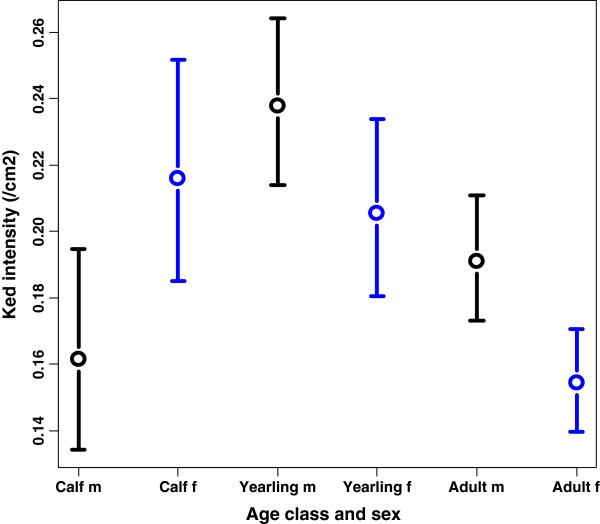
**Predicted deer ked intensity (mean ± SE) by sex and age class in moose, at average values of covariables from best model.** Male (m) and female (f) moose in the following age classes; calf (~ 0.5 years), yearlings (~1.5 years) and adult (> 2.5 years).

Our best model predicted highest ked intensity in male yearlings, higher than both male calves and bulls (p = 0.06 and 0.09, respectively), while cows displayed lowest ked intensity, significantly less than both female calves and female yearlings (Table 2; Figure 2). Adult bulls had higher ked intensities than cows (p = 0.07), while insignificant differences were found between sexes in calves and yearlings. Ked intensity in male yearlings was estimated to be 0.238 keds/cm^2^ at average level of all continuous covariables.

Moose population density tended to be positively correlated with ked intensity (Table 2). Our model predicted about 9% increase in ked intensity (0.238 to 0.260 keds/cm^2^) in male yearlings if moose density increased with 0.25 units from average (0.497 to 0.747 “moose seen per hunter day”, which is in the upper quartile of moose density).

Ked intensity tended to decrease with increasing altitude (Table 2). For example, about 12% decrease in ked intensity in male yearlings (0.238 to 0.210 keds/cm^2^) was predicted if altitude increased 100 meters from average (249 to 349 m.a.s.l., which is in the upper quartile of altitude).

Land cover of Scots pine, and to a lesser extent coniferous forest, around killing sites was strongly linked to high ked intensity (Table 2). Scots pine dominated in the western part of the study area (Figure 1). An increase of about 28% in ked intensity in male yearlings (0.238 to 0.304 keds/cm^2^) was predicted if land cover of Scots pine increased from 33.2% (average) to 58.2% (upper quartile of Scots pine). Similarly, a 22% reduction (0.238 to 0.186 keds/cm^2^) in ked intensity was predicted if land cover of Scots pine decreased from 33.2% (average) to 8.2% (lower quartile of Scots pine). Despite representing a small proportion of the total land cover, the cover of bogs was negatively associated with ked intensity in the study area (Table 2).

Increased longitude, as opposed to increased latitude, was positively associated with ked intensity (Table 2). An increase of about 30% in ked intensity in male yearlings (0.238 to 0.310 keds/cm^2^) was predicted if longitude increased 30 minutes from average (11°45``E to 12°15``E). The inclusion of a 2^nd^ order term for latitude suggested a non-linear relationship (Table 2). Ked intensity was quite high at low latitude, but a gradual decrease (0.238 to 0.0960 keds/cm^2^) was predicted from 60°00``N (average) to 60°15``N (northern edge), representing about 60% reduction in ked intensity.

## Discussion

Ectoparasites causes harm to animals and humans and may spread disease. With ongoing climate change, affecting the life cycles and distribution ranges of many parasites [2], and the increase in population density of many large ungulates in Europe [27], there is an urgent need to understand what causes variation in prevalence and infestation intensity. Our results revealed a deer ked infestation prevalence of 100%, similar to that Paakkonen *et al*. [28] report from Finnish moose. Despite different histories of colonization [6] and a higher density of definitive hosts in Norway compared to Finland [8], the deer ked is apparently an ectoparasite well-adapted to Fennoscandian moose and seems able to exploit the entire moose population within its distribution range. The generality of our results are further supported by the 100% deer ked infestation prevalence reported from Polish moose examined in 1988 [29].

### Ked infestation intensity in relation to moose age and gender

Although all moose were infested with keds, a wide range in deer ked intensity (0.004 – 1.405 keds/cm^2^) was observed, with the highest intensity in male yearlings (Figure 2). Our study does not reveal the mechanism behind this wide range, but differences between age and sex classes may be explained by physical and behavioral differences. Firstly, Kadulski [30] and Kortet *et al.*[31] argue that swarming keds prefer large body size when choosing hosts. As calves follow their mother during their first autumn this may imply that cows will attract higher numbers than their accompanying calves. Hence, calves at foot may partly be protected against deer ked attacks. However, body size alone cannot explain the pattern, since yearlings had higher infestation than adults. Secondly, moose calves are born with a reddish coat and their moulting period is July to September, in contrast to adults moulting between April and July [32]. Hence, deer ked swarming and calves moulting coincides, which most likely hamper deer ked establishment in the calves` coat. Kortet *et al*. [31] did not find any difference in ked preferences for black and red, mimicking cow and calf colors, stating that coat color is not important for ked host selection. Thirdly, locally acquired resistance to sheep keds (*Melophagus ovinus*) was demonstrated in an experiment with lambs (*Ovis aries*) [33]. Resistance was gradually lost over the following weeks after infestation, but the experiment demonstrated that repeated infestation in the same test area reduced time to onset of resistance [33]. This suggests that adult moose exposed to several swarming seasons may develop resistance more quickly than yearlings, resulting in higher ked intensity in the latter age class, fitting our results. Fourthly, calves are less active than yearlings, cows without calves and especially bulls, during autumn. Kortet [31] argues that movement is the main cue in ked host selection and therefore calves might be less exposed to winged keds sitting in the vegetation, waiting to flying onto any moving object passing by. In a closely related study in Finland, Paakkonen *et al.*[28] stated that bulls had about three times the intensity of keds compared to cows. Our model also predicted significantly higher ked intensity in bulls compared to cows, but the sex difference was less pronounced (Figure 2). Heavier infestation in bulls in autumn is consistent with expectations from the life history theory as a tradeoff may exist between resources required for parasite resistance and reproduction. Accordingly, parasite levels are found to increase in males during the rutting season [34].

During close physical contact between cow and calf, direct transfer of wingless keds may also affect the infestation rate. Small [35], citing Tetley 1958, states that sheep keds migrate to the surface of the fleece in response to increased ambient temperature. Deer keds displayed similar migrating behavior from skin surface to the tip of hair when moose pelts were brought from subzero temperature into a heated room (20°C) (unpublished, Madslien), indicating that transfer of deer keds can occur between moose in close physical contact. Similarly, Davis *et al*. [36] and Samuel and Trainer [37] showed that Neotropical deer ked (*Lipoptena mazamae*) could be transferred from doe to fawn in white tailed deer (*Odocoileus virginianus*). However, we regard it likely that direct transfer is relatively small compared to direct settlement of winged keds.

### Effect of habitat on infestation intensity

The preferred habitats for pupal development and survival, as well as winged imagines host acquisition, are discussed in the literature [38]. In our study, we expected to find a positive correlation between preferred moose habitats during autumn and winter, i.e. areas where the deer ked pupae will be deposited during the reproductive season of this insect, and deer ked intensity. Our study indicates that moose living, or at least shot, in a habitat dominated by Scots pine, an important species for moose browsing during late autumn and winter, had the highest infestation loads, hence consistent with our first hypothesis. Haarløv *et al.*[38] argued that puparia and winged imagines prefer woody areas, due to the soils loose structure, moderate moist and limited wind all year round. Haarløv *et al.* also found that red deer (*Cervus elaphus*) had higher deer ked intensity than fallow deer (*Dama dama*) and explained the difference with habitat preferences. Fallow deer prefer open grasslands, where pupae that drop from the coat will be exposed to extreme and possibly fatal conditions, whereas red deer favor more protected woody areas. In our model, bogs were negatively correlated with ked intensity, possibly explained by adverse conditions for pupal survival in humid substrates or because these areas are avoided by moose due to their low production of feed. Samuel *et al.*[37] speculated that flooding in lowlands, resembling the condition in a bog, killed a high number of soil-dwelling Neotropical deer keds in Texas, which substantiates the notion that very damp soil has a negative impact on pupal development and survival. Lack of high vegetation as vantage points for winged keds searching for hosts and being exposed to heavy winds may also prevent bogs from being a suitable habitat for winged keds. On the other hand, Darling *et al.*[39] explained high intensity of keds at wallows with the rubbing behavior of red deer during moulting in April and May, resulting in a large number of pupae released from the coat simultaneously. If this is the case in adult moose, which are moulting in early summer [32], preferred habitats during this period should have an increased deer ked intensity. In this part of the year, moose are typically feeding on emerging deciduous leaves in deciduous and young spruce forests [40]. Hence, the lack of any association between infestation intensity and the coverage of spruce or deciduous forest lend little support to this hypothesis in our study.

### Effect of moose population density on infestation intensity

Because moose is the preferred definitive host of deer keds in Norway, we hypothesized a strong, positive correlation between moose population density and deer ked intensity, as Balashov [10] reported from moose in northwestern Russia. Balashov [10] monitored human deer ked infestation intensities in three different geographical areas for 8 consecutive years and observed on average a 8 to 29 fold decrease in ked intensity from 1991 to 1995. During this period, a corresponding dramatic reduction in moose population density was claimed to be the cause of decreased ked attacks on human study objects walking in the forest. A similar tendency, although weaker, was detected in our model (Table 2), hence partly supporting our second hypothesis. However, the observation of an association weaker than expected might have been influenced by little variation in moose population density (average; 0.50 moose seen per hunter-day, SD ± 0.20, range; 0.15 to 1.21). Moreover, as density was measured by its proxy moose seen per hunting effort, there is also a chance that varying habitat composition and hunting methods may have affected the precision of this variable. Given our current results, however, it is unlikely that a small regulation of moose numbers by managers will cause large effects on deer ked infestation.

Although not included in our study, seasonal migration by moose can be expected to affect deer ked intensity in other parts of Scandinavia. Moose in the study area are mostly stationary [23], contrary to populations further north and west, where a varying part of the population migrate between summer areas at higher latitudes and low altitude winter areas. Because pupae are mainly shed in the winter areas and swarming occurs in the summer area, only a proportion of the population will be exposed to keds during the swarming season. Hence, high deer ked intensities in stationary moose could partly be attributed to an accumulation of keds in moose habitats utilised throughout the year and the fact that the entire moose population is exposed to keds during the swarming season.

### Effect of latitude, longitude and altitude on infestation intensity

Our best model predicted a negative association between ked intensity and latitude and a positive association between ked intensity and longitude (Table 2), supporting our third hypothesis of a decreasing gradient in ked intensity from southeast to northwest. Härkönen *et al*. [41] also demonstrated decreasing off-host survival and pupal development of keds towards higher latitudes, but explained the results by reduced summer temperatures along an 1000 km long geographical gradient. In our study, the geographical gradient in latitude is only 70 km, which means that alterations in temperature within the study area due to difference in latitude alone are not likely to occur. As an alternative, we suggest that the decreasing ked intensity from southeast to northwest is due to the keds` main direction of dispersal and the possibility that the parasite population has not yet reached the carrying capacity in the whole study area.

Altitude in the study area ranges from about 100 to 466 m a.s.l. and killing sites were found between 111 and 455 m a.s.l, indicating that moose utilize habitats in the entire range of elevation gradients. A tendency of negative correlation between ked intensity and altitude was supported in our data (Table 2). Kovanci *et al*. [42] explained a negative correlation in cherry fruit fly (*Rhagoletis cerasi*) intensity and altitude by about 0.5°C decrease in temperature per 100 m increase in altitude and not by the elevated altitude *per se*. Since temperature is regarded as an important factor for off-host survival and pupal development of deer keds [41,43], an indirect effect of altitude, through decreasing temperature, may explain the effect of altitude in our data. However, we cannot exclude the possibility of a slight contraction of moose space use from higher to lower altitudes during winter (e.g. due to more favorable snow conditions). Hence, the higher ked intensity may also be a result of more pupae being shed at lower altitudes.

#### Methods (sampling period, anatomical site and procedures)

We chose to restrict sampling to the first week of the hunting season for two reasons. First, we know from experience that about half of the total numbers of moose are shot during the first week of hunting, hence this period was best to maximize sample size. Moreover, allowing a longer sampling period than a week may bias the intensity results by a time-dependent, cumulative effect of swarming keds attaching to the coat.

Previous studies in moose [9,28] and red deer [38] have shown that deer keds are distributed all over the hide, with aggregates around the axilla-neck and groin-anal regions. Neck region, in contrast to the groin, is covered by long guard hairs which protect keds from being torn out of the coat, both during moose movement through dense vegetation and post-killing transport and handling by humans. Hence, sampling from the dorsal neck region of hunted moose was chosen.

Three methods of quantitative collection of deer keds (*L.cervi* and *L.mazamae*) in the coat are described in the literature; cutting the hair coat with scissors [28], combing [36] and digestion with KOH [44]. We developed our own method by removing the coat with a wool clipper, followed by careful inspection of hair and naked skin for keds.

In hindsight, this method was suboptimal due to the risk of cutting parasites into pieces, and hence biasing the number of keds per sample. To avoid this, we only counted the anterior part of demolished keds.

Welch *et al.*[45] found that random sampling of 15% of a moose hide total area is sufficient to estimate densities of winter ticks (*Dermacentor albipictus*). Similar studies are not performed with deer keds, but using this study as a guideline, we inspected about 13.4% (20 × 20 cm = 400 cm^2^/29.833 cm^2^ average skin area for adults [28]) of total skin area in adults and 21.4% (400 cm^2^/18.666 cm^2^[28]) average skin area for calves. Based on Welch`s [45] study, the size of our skin samples were sufficient for estimating total ked densities in calves, but somewhat small for the same estimation in adults.

Since unfavorable weather conditions [46], habitat [38] and predation [13,14] may affect emergence success of pupae, survival of pupae through winter, spring and summer is important for ked abundance. However, within its core distribution range, pupal survival rates are assumed to be high [41] and therefore we did not include proxies of pupal survival as variables in our study.

## Conclusions

Abundant deer ked swarming is considered as a major obstacle for human outdoor activity during autumn and they are known to be vectors of pathogenic bacteria. A recent study isolated *Bartonella* spp. in both deer keds and moose blood [47], indicating that moose most likely represents a reservoir of infection and that keds acts as a vector for spread of infection with *Bartonella* spp. Further, a recent epizootic of alopecia in moose was associated with massive deer ked infestation [9] and the probable harassment for domestic animals should not be neglected. Our study reveals that deer keds are ubiquitous in moose within its distribution range in southeastern Norway, but individual infestation intensities vary substantially. This is possibly due to differences in behavior, body size and resistance between age classes and the sexes in the moose population. Ked intensity only tended to be positively associated with moose density within the fairly narrow variation in moose density within the study area, suggesting managers cannot expect a large effect on the keds with only a minor regulation in moose numbers. However*,* since the main hunting period is after swarming, a moose management strategy targeting harvest of calves (by not allowing them to become yearlings) and keeping the moose population on a relatively low level (since all moose are infested) seems advisable for reducing both deer ked intensity and potential risk of pathogen transmission from keds to humans and animals.

## Competing interests

The authors affirm that they have no competing interests.

## Authors’ contributions

KM, EJS, AM and BY designed the study. KM and KRB performed field work. KM, KRB performed laboratory examination. KM, HV, AM, EJS and BY did data analysis and interpretation. KM and BY drafted the manuscript. KM, EJS, AM, BY, HV and KRB revised the manuscript. All authors approved the final version for submission.
